# Supplementary material for: GIV/Girdin, a non-receptor modulator for Gαi/s, regulates spatiotemporal signaling during sperm capacitation and is required for male fertility
Source: eLife. 2021 Aug 19;10:e69160. doi: 10.7554/eLife.69160 (PMC8376251; doi:10.7554/eLife.69160)
Supplement: Figure 1—source data 2. [file elife-69160-fig1-data2.pptx]

## Slide 1
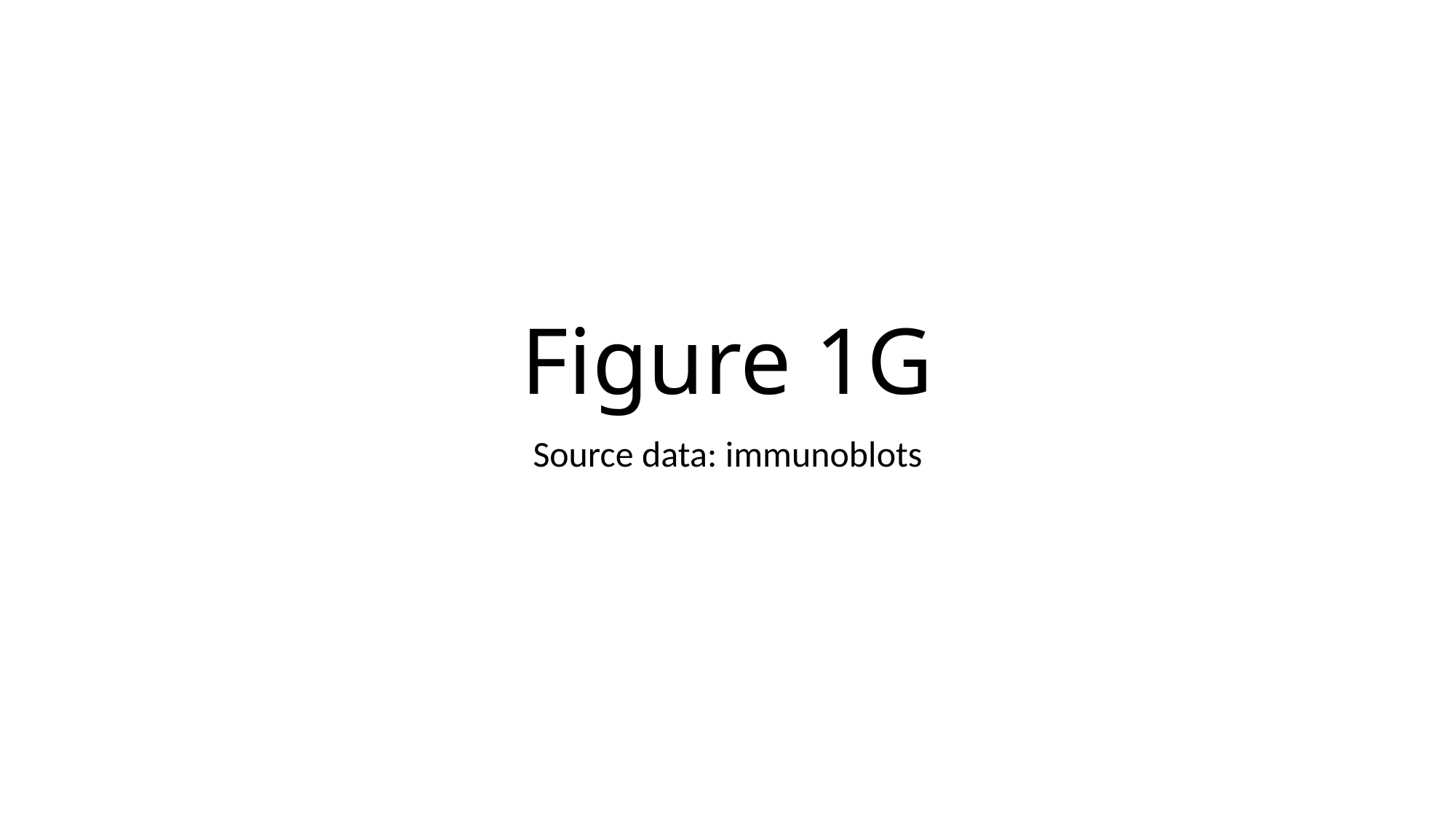

# Figure 1G
Source data: immunoblots

## Slide 2
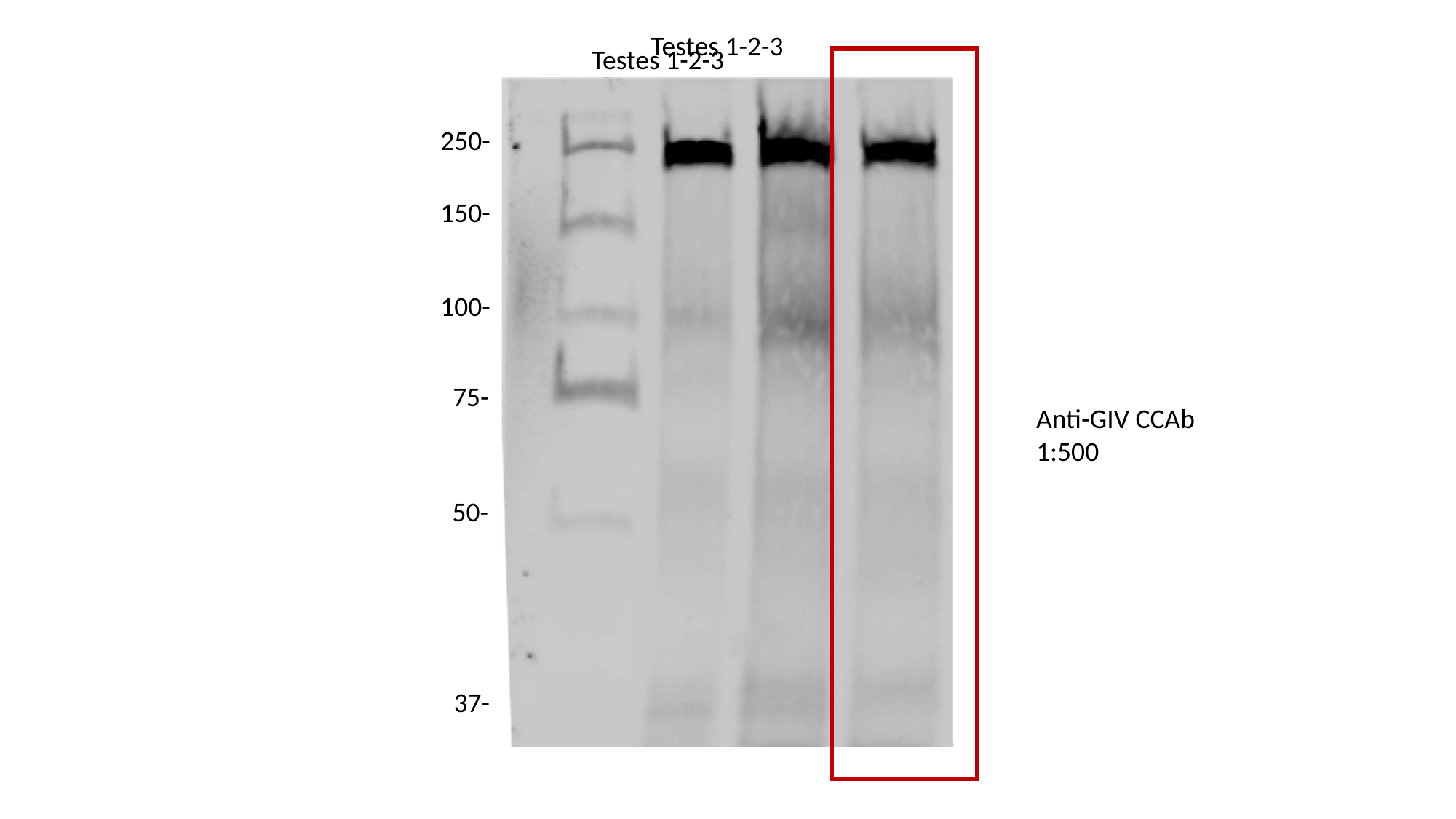

Testes 1-2-3
Testes 1-2-3
250-
150-
100-
75-
Anti-GIV CCAb
1:500
50-
37-

## Slide 3
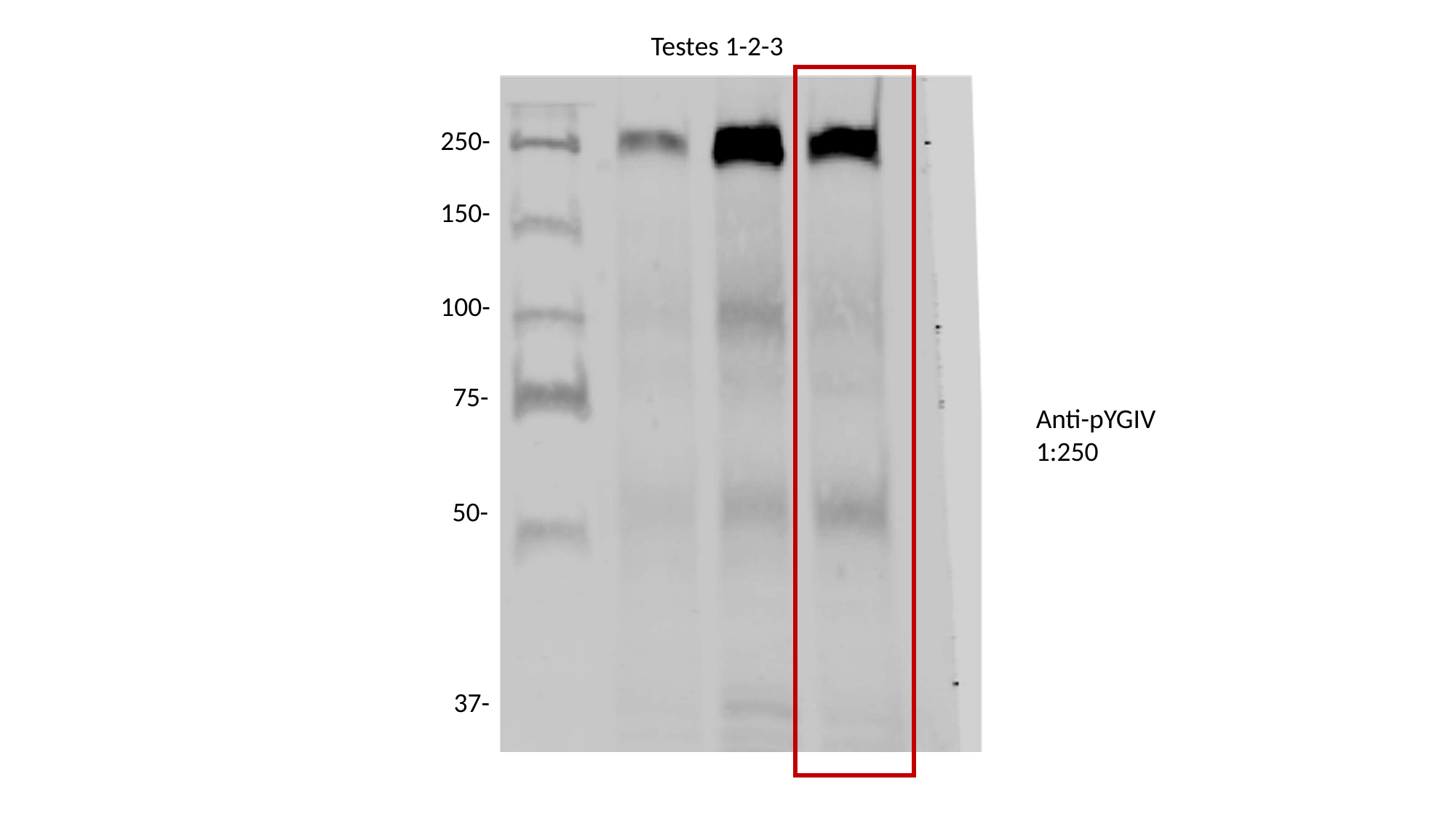

Testes 1-2-3
250-
150-
100-
75-
Anti-pYGIV
1:250
50-
37-
