# Supplementary figures and images for: GIV/Girdin, a non-receptor modulator for Gαi/s, regulates spatiotemporal signaling during sperm capacitation and is required for male fertility
Source: eLife. 2021 Aug 19;10:e69160. doi: 10.7554/eLife.69160 (PMC8376251; doi:10.7554/eLife.69160)

## Slide 1
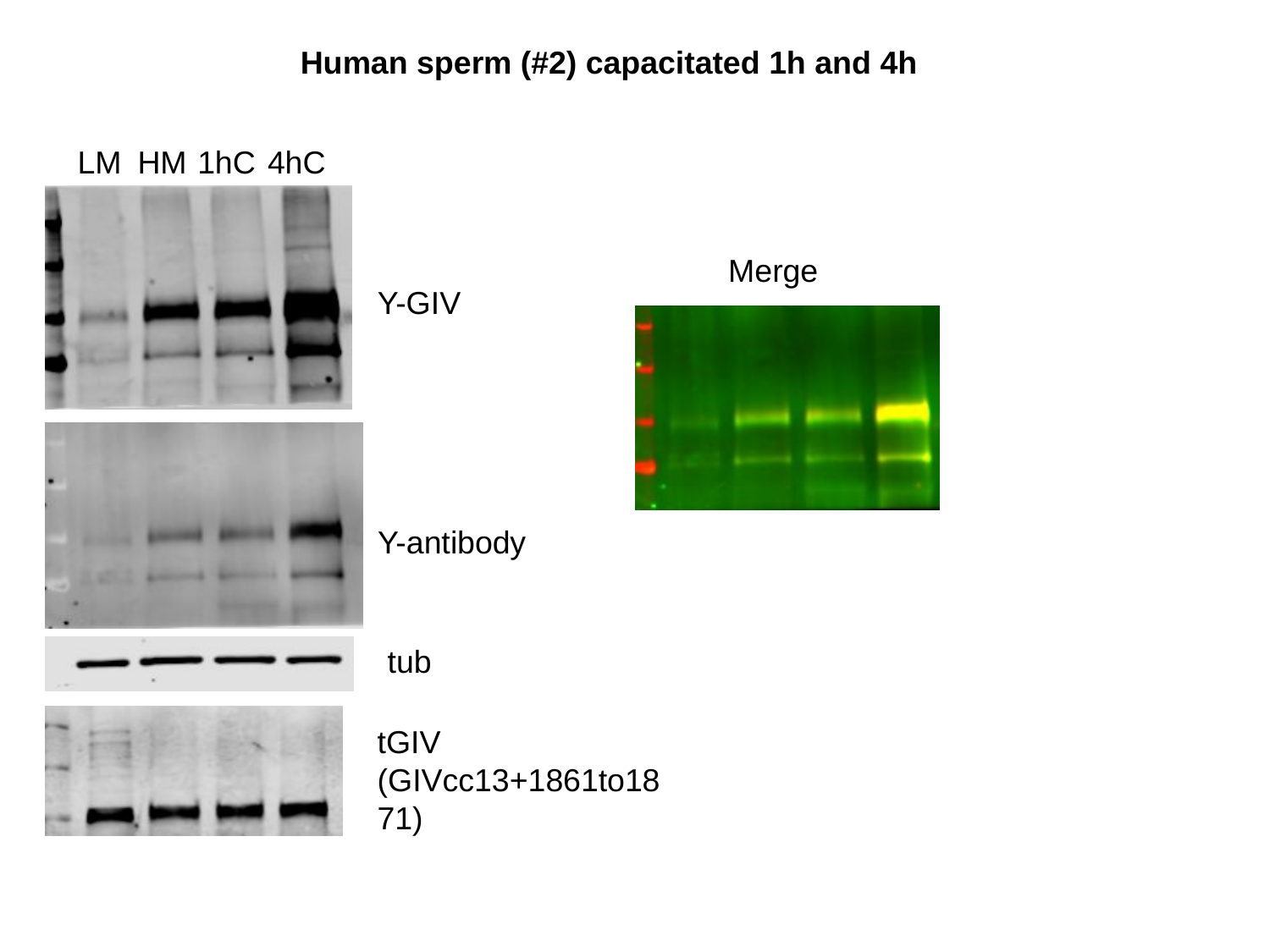

Human sperm (#2) capacitated 1h and 4h
LM
HM
1hC
4hC
Merge
Y-GIV
Y-antibody
tub
tGIV (GIVcc13+1861to1871)

Supplement: Figure 3—source data 1. [file elife-69160-fig3-data1.pptx]

## Slide 1
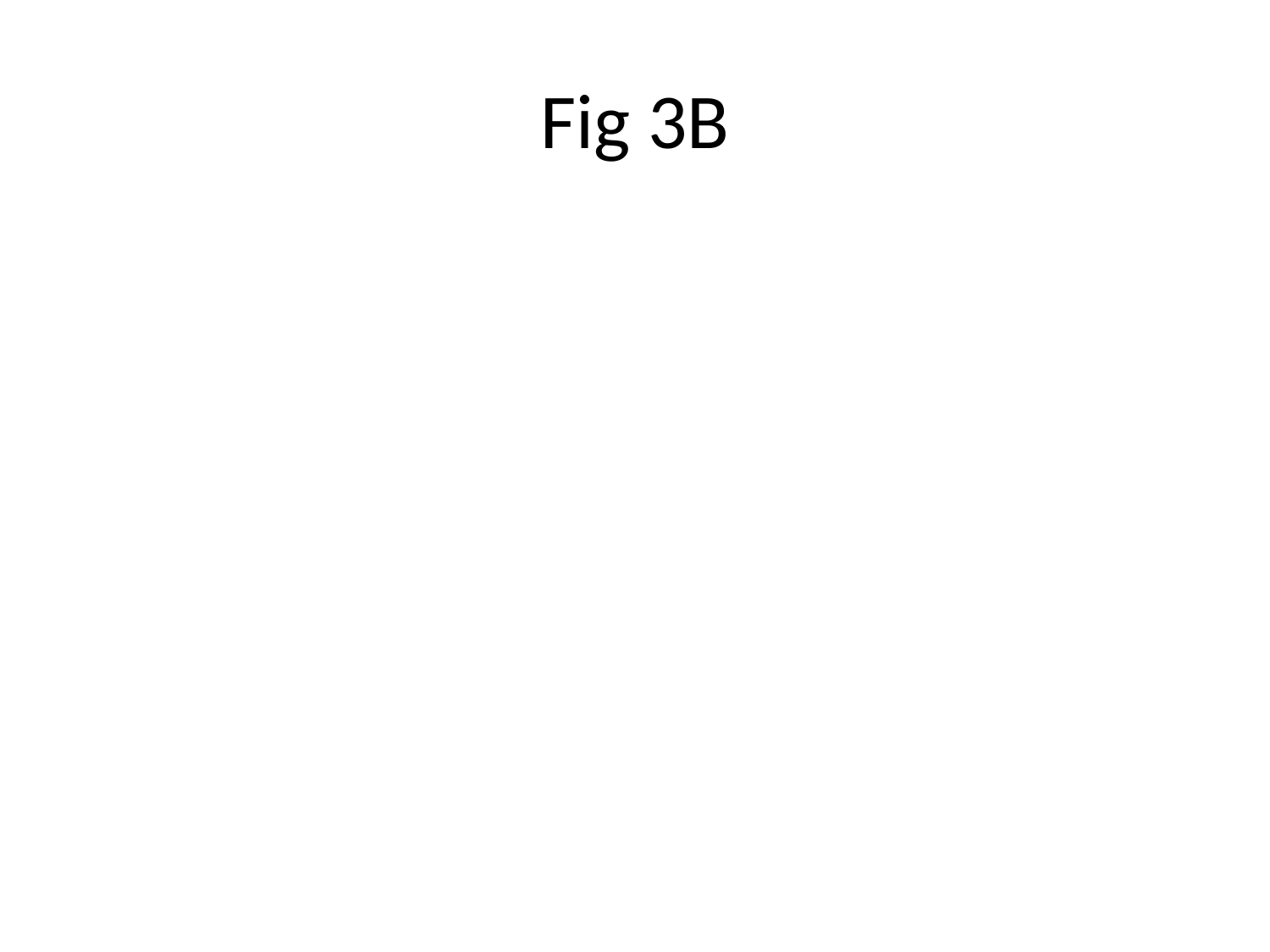

# Fig 3B

## Slide 2
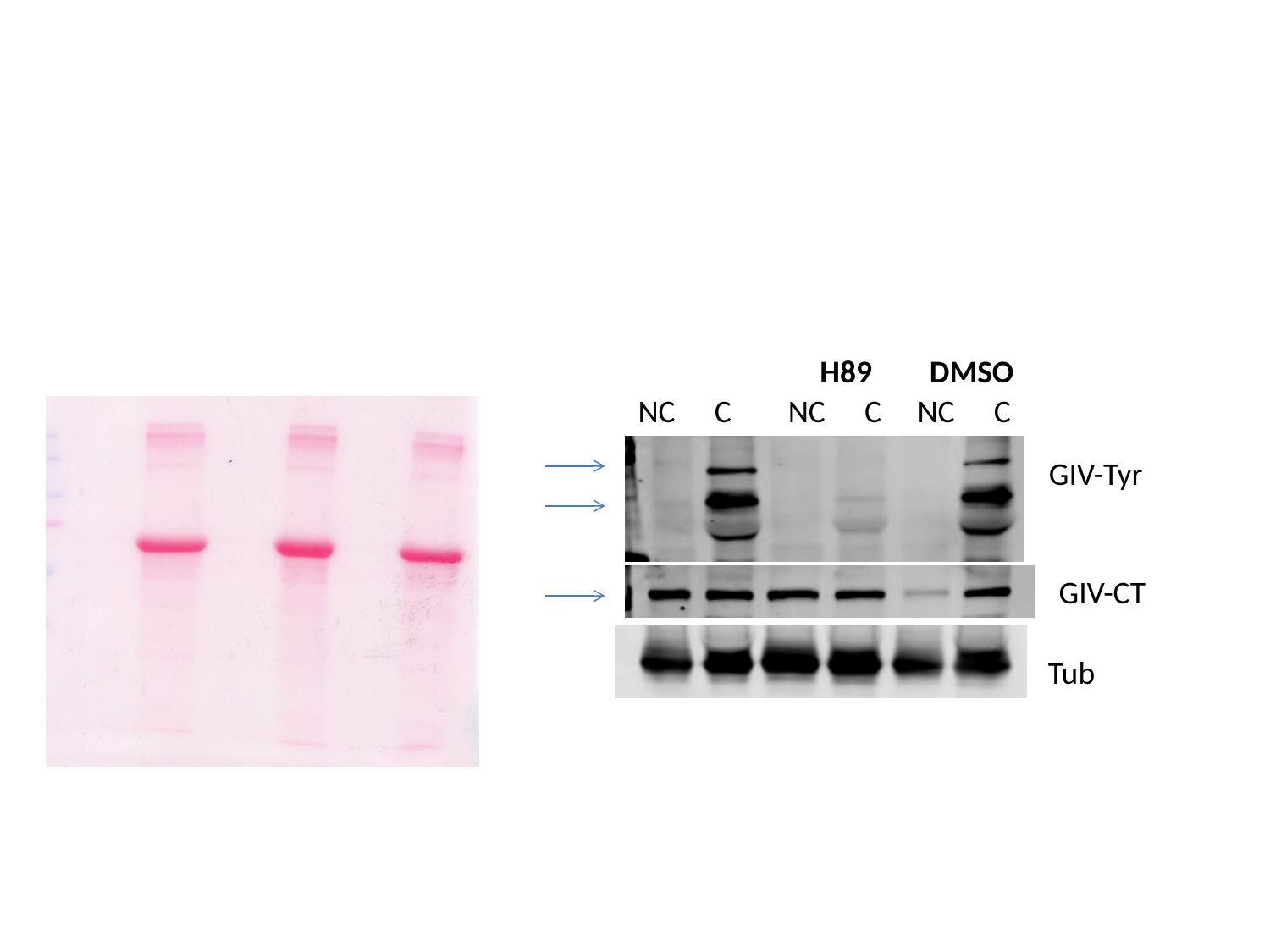

H89
DMSO
NC
C
NC
C
NC
C
GIV-Tyr
GIV-CT
Tub

Supplement: Figure 3—source data 2. [file elife-69160-fig3-data2.pptx]
