# Supplementary material for: GIV/Girdin, a non-receptor modulator for Gαi/s, regulates spatiotemporal signaling during sperm capacitation and is required for male fertility
Source: eLife. 2021 Aug 19;10:e69160. doi: 10.7554/eLife.69160 (PMC8376251; doi:10.7554/eLife.69160)
Supplement: Figure 3—source data 3. [file elife-69160-fig3-data3.pptx]

## Slide 1
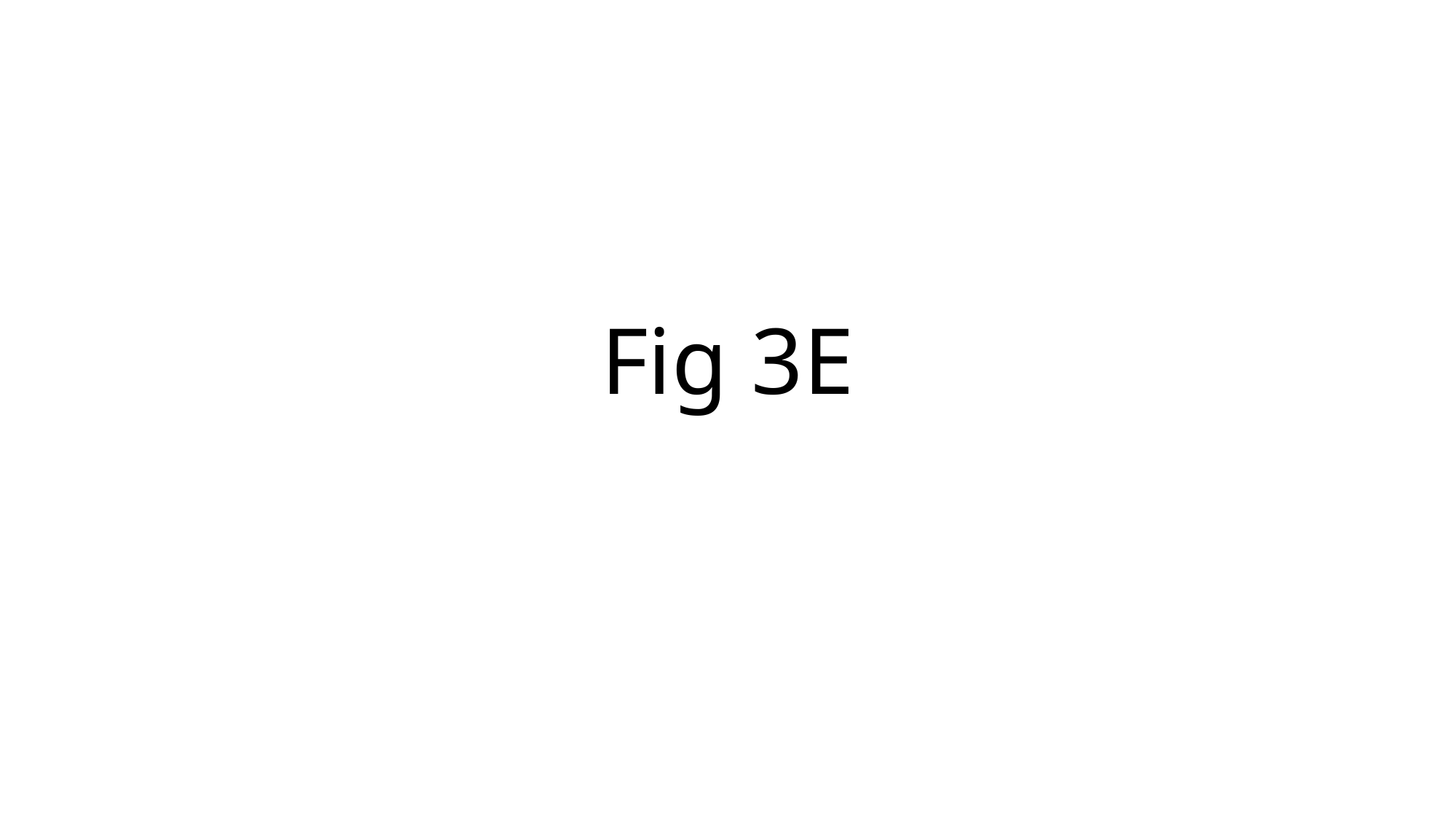

# Fig 3E

## Slide 2
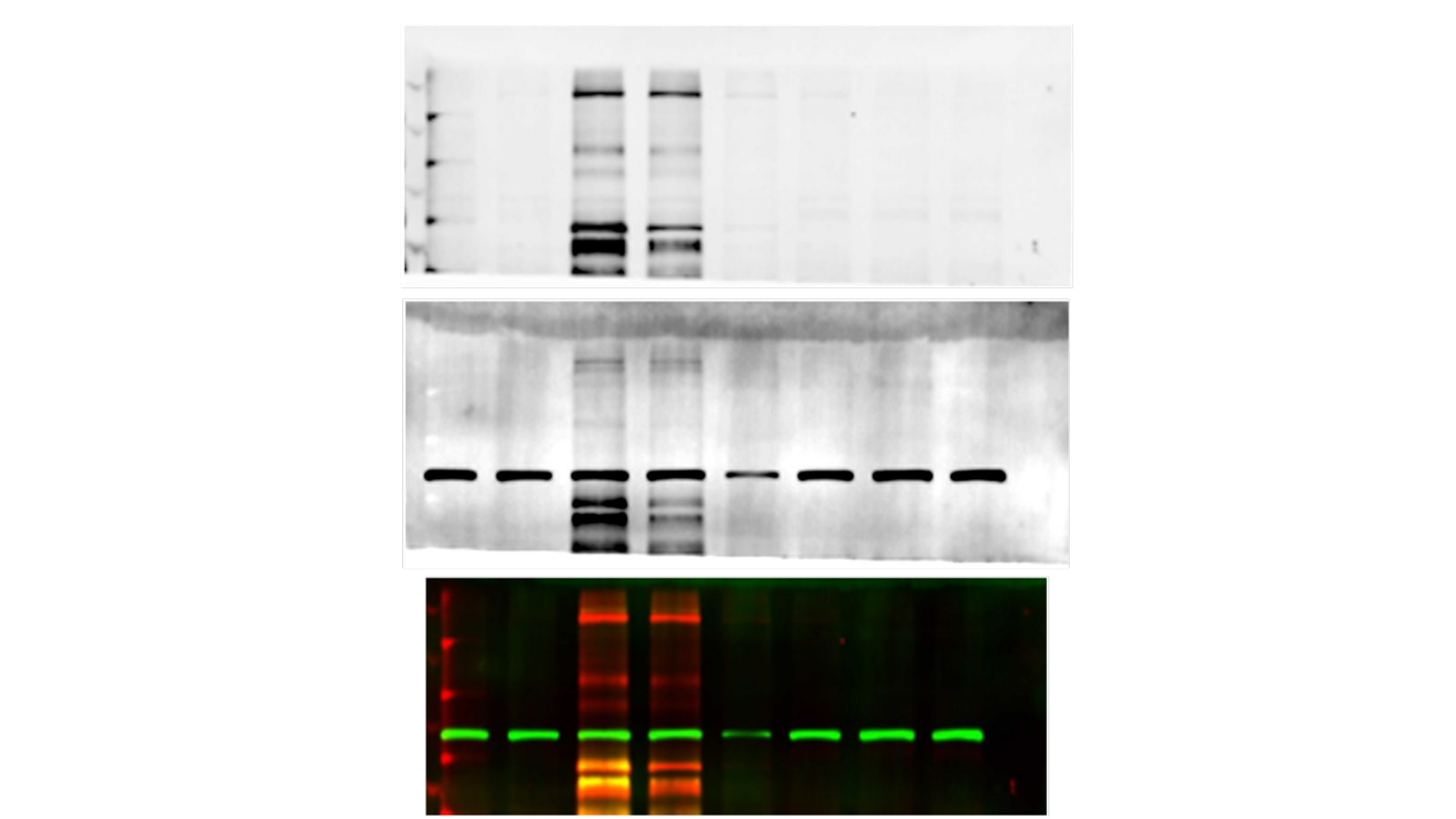

## Slide 3
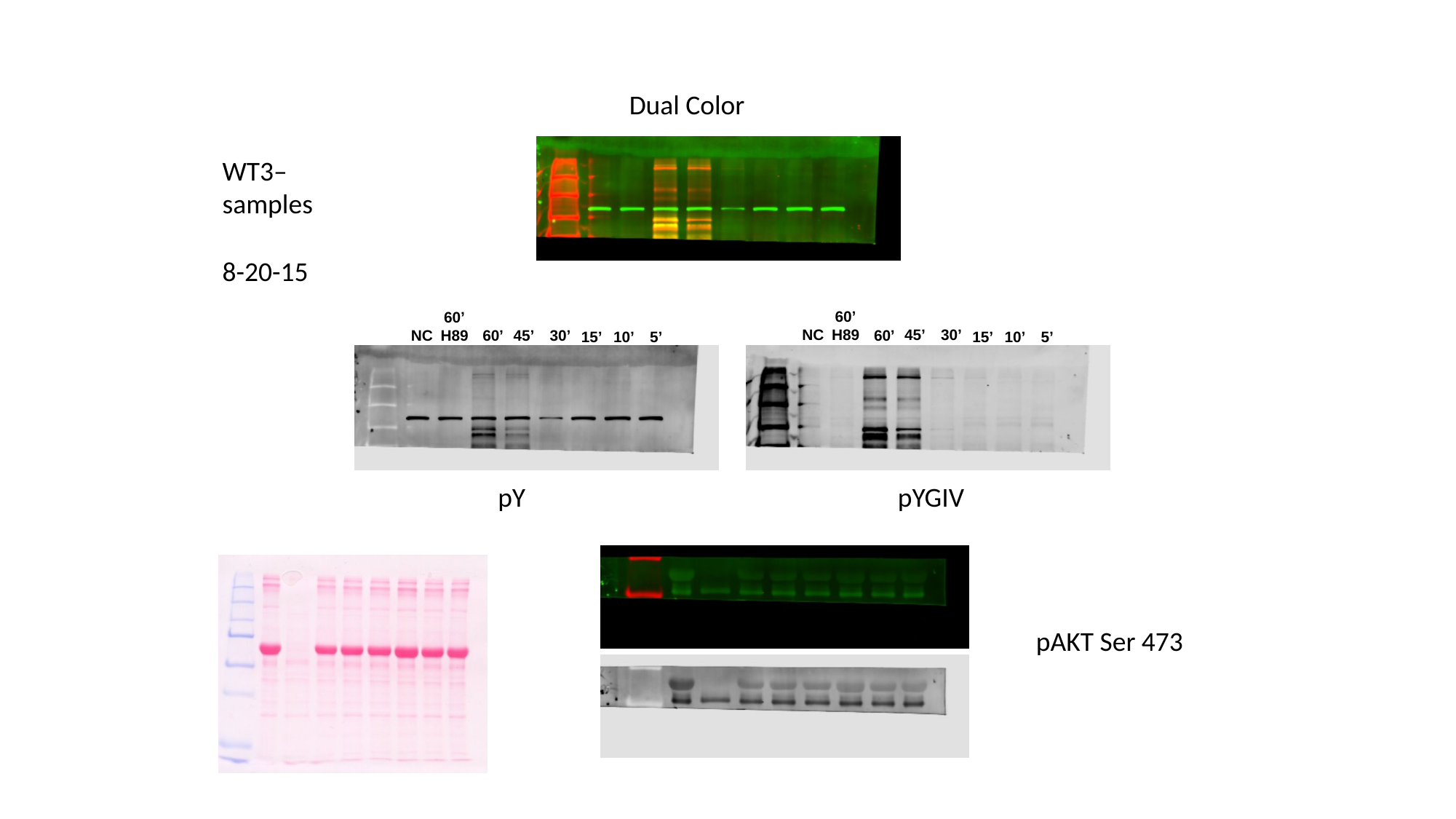

Dual Color
WT3–
samples
8-20-15
60’
H89
NC
45’
30’
60’
15’
10’
5’
60’
H89
NC
45’
30’
60’
15’
10’
5’
pY
pYGIV
pAKT Ser 473

## Slide 4
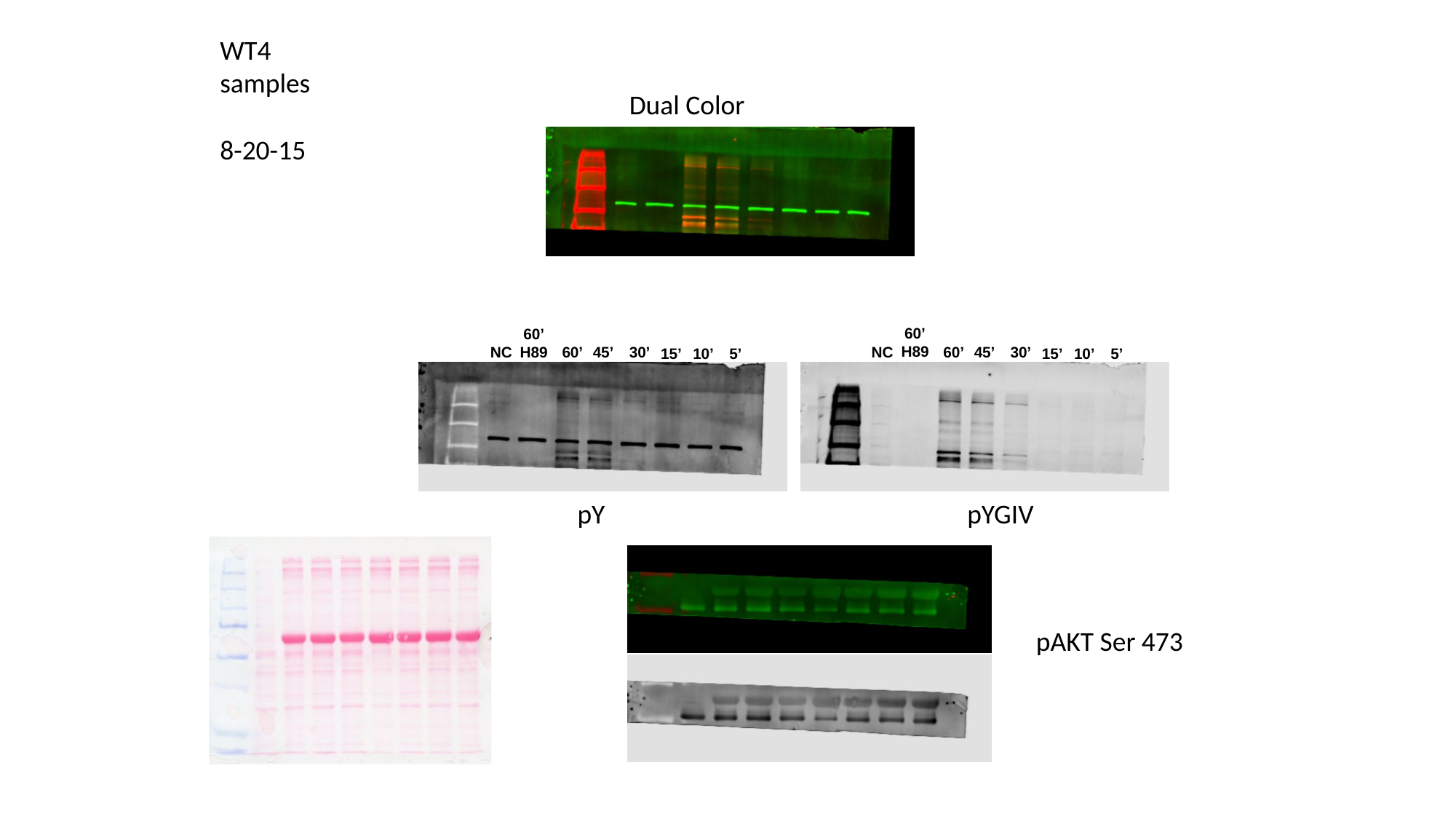

WT4
samples
Dual Color
8-20-15
60’
H89
NC
45’
30’
60’
15’
10’
5’
60’
H89
NC
45’
30’
60’
15’
10’
5’
pY
pYGIV
pAKT Ser 473

## Slide 5
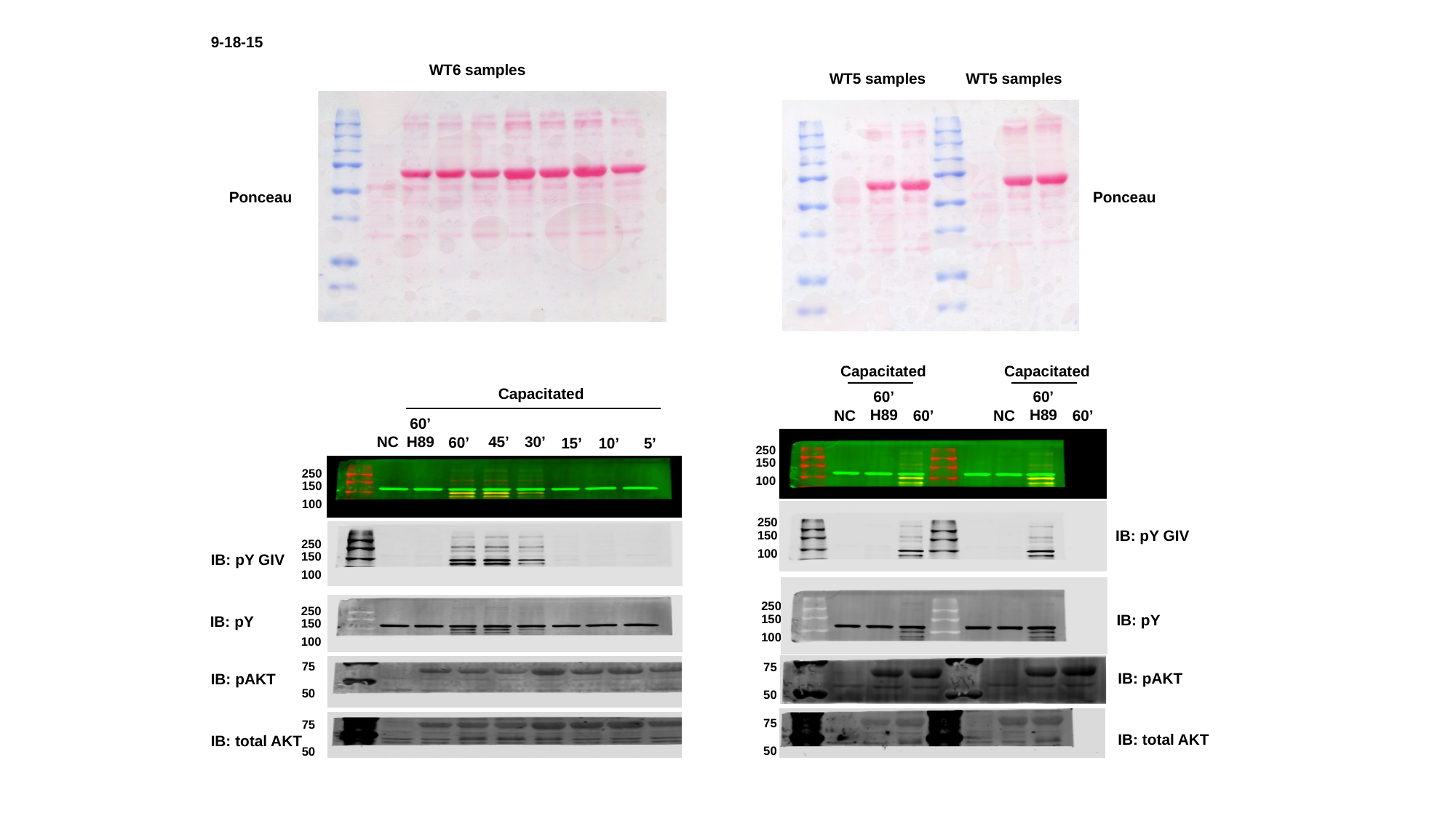

9-18-15
WT6 samples
WT5 samples
WT5 samples
Ponceau
Ponceau
Capacitated
Capacitated
Capacitated
60’
H89
NC
60’
60’
H89
NC
60’
60’
H89
NC
45’
30’
60’
15’
10’
5’
250
150
100
250
150
100
250
150
100
IB: pY GIV
250
150
100
IB: pY GIV
250
150
100
250
150
100
IB: pY
IB: pY
75
50
75
50
IB: pAKT
IB: total AKT
75
IB: pAKT
50
75
IB: total AKT
50
