# Supplementary material for: GIV/Girdin, a non-receptor modulator for Gαi/s, regulates spatiotemporal signaling during sperm capacitation and is required for male fertility
Source: eLife. 2021 Aug 19;10:e69160. doi: 10.7554/eLife.69160 (PMC8376251; doi:10.7554/eLife.69160)

## Slide 1
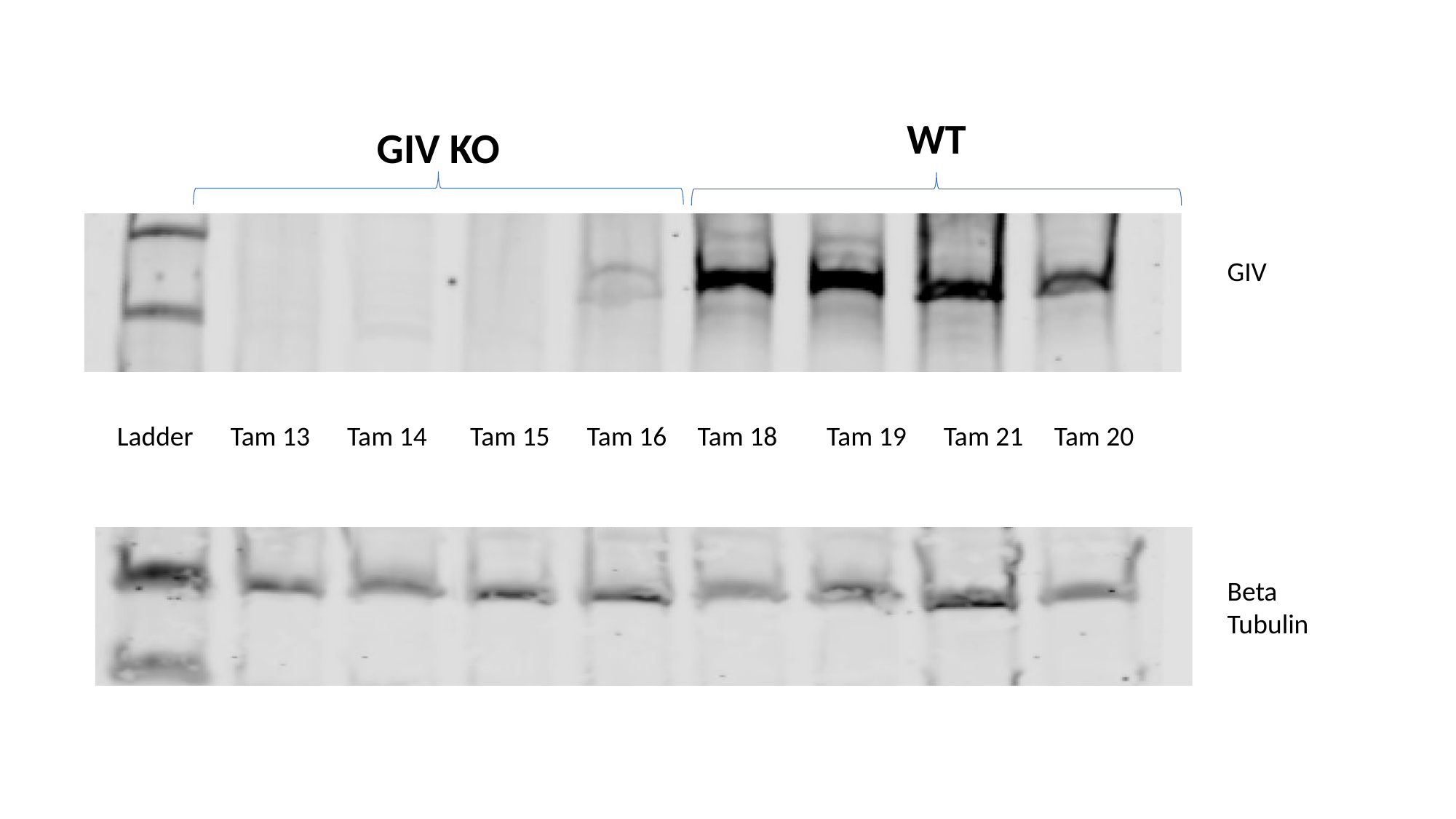

WT
GIV KO
GIV
Ladder Tam 13 Tam 14 Tam 15 Tam 16 Tam 18 Tam 19 Tam 21 Tam 20
Beta Tubulin

## Slide 2
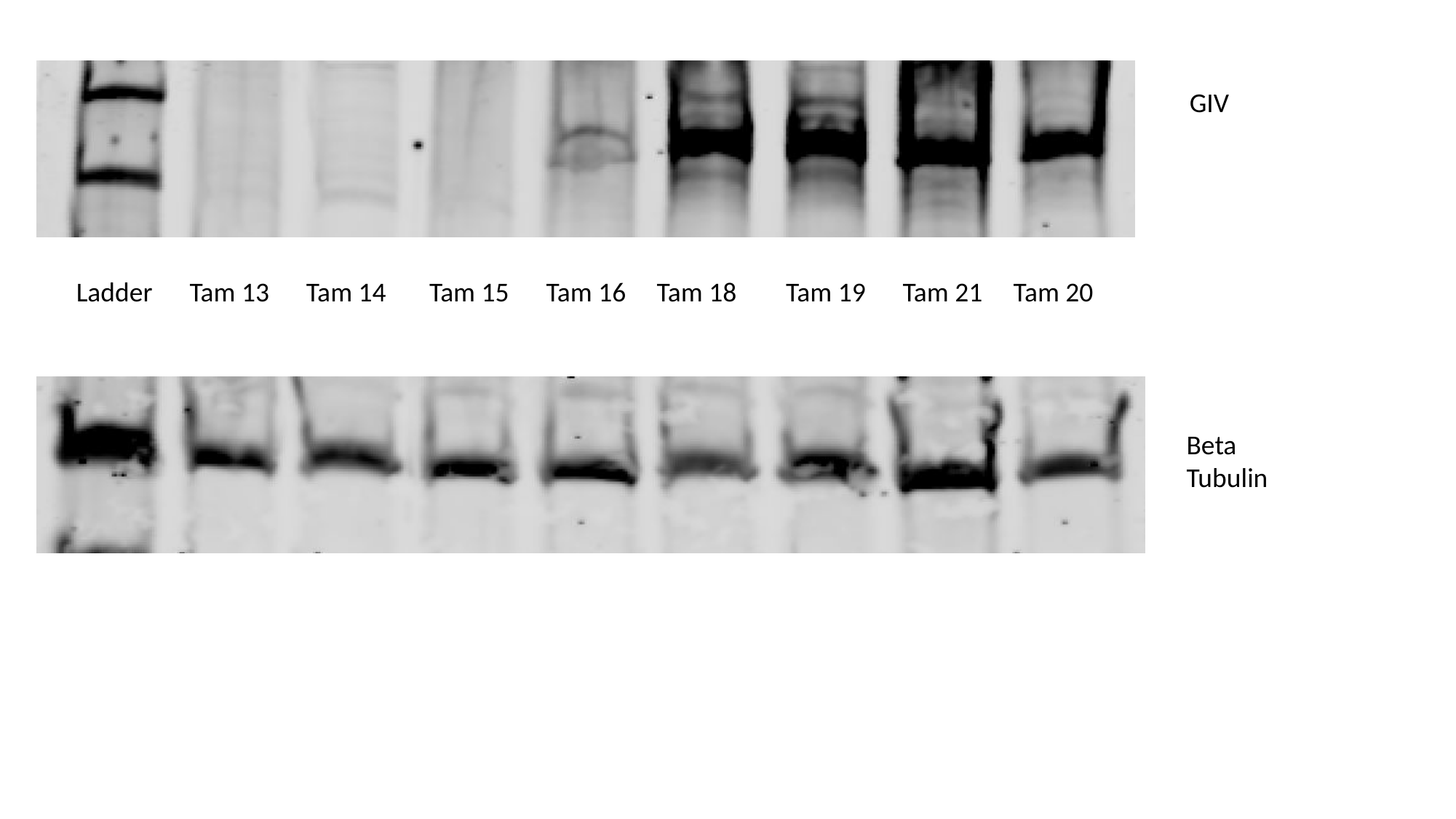

GIV
Ladder Tam 13 Tam 14 Tam 15 Tam 16 Tam 18 Tam 19 Tam 21 Tam 20
Beta Tubulin

## Slide 3
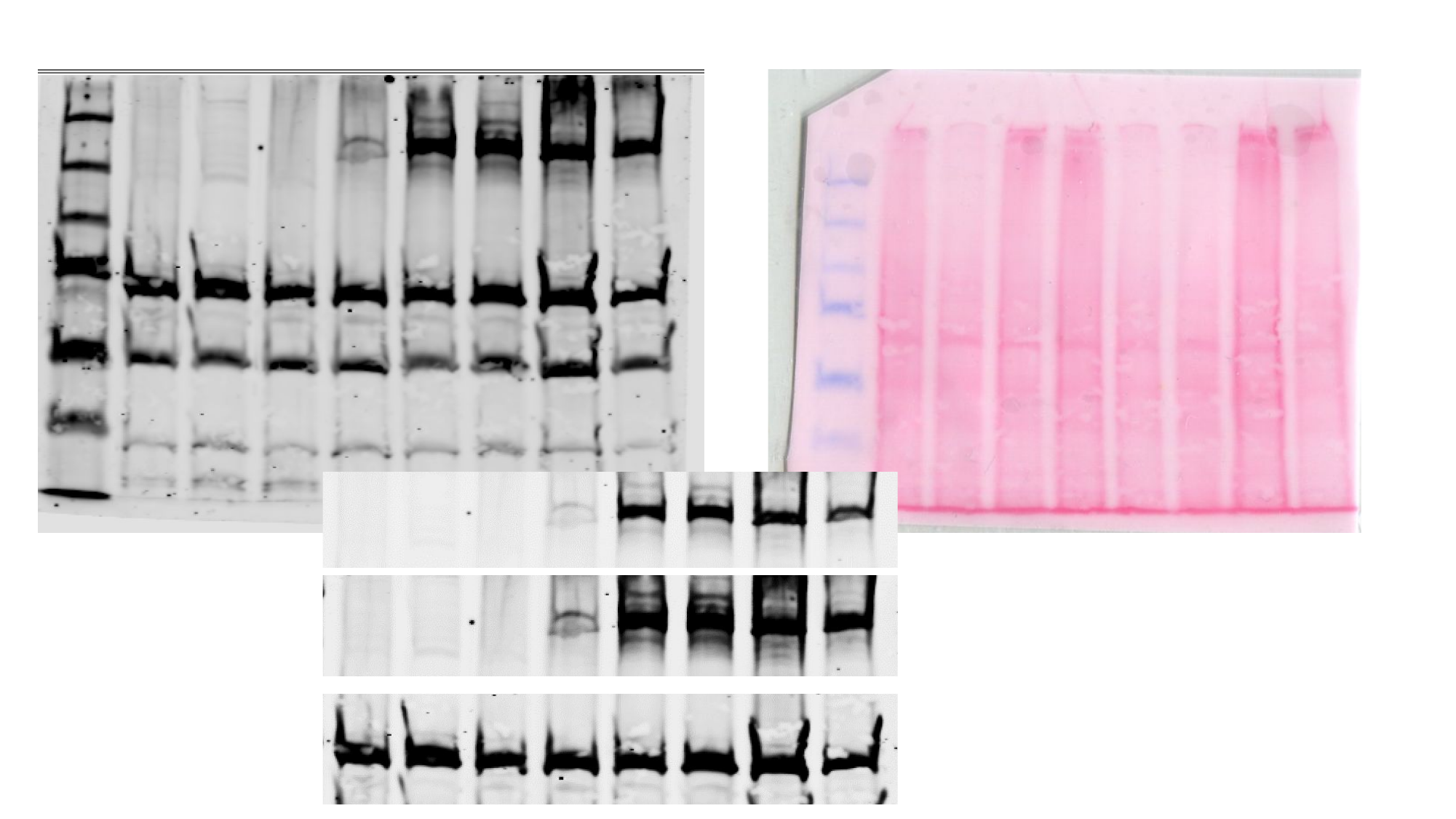

Supplement: Figure 5—source data 1. [file elife-69160-fig5-data1.pptx]
