# Supplementary material for: GIV/Girdin, a non-receptor modulator for Gαi/s, regulates spatiotemporal signaling during sperm capacitation and is required for male fertility
Source: eLife. 2021 Aug 19;10:e69160. doi: 10.7554/eLife.69160 (PMC8376251; doi:10.7554/eLife.69160)
Supplement: Figure 6—source data 1. [file elife-69160-fig6-data1.pptx]

## Slide 1
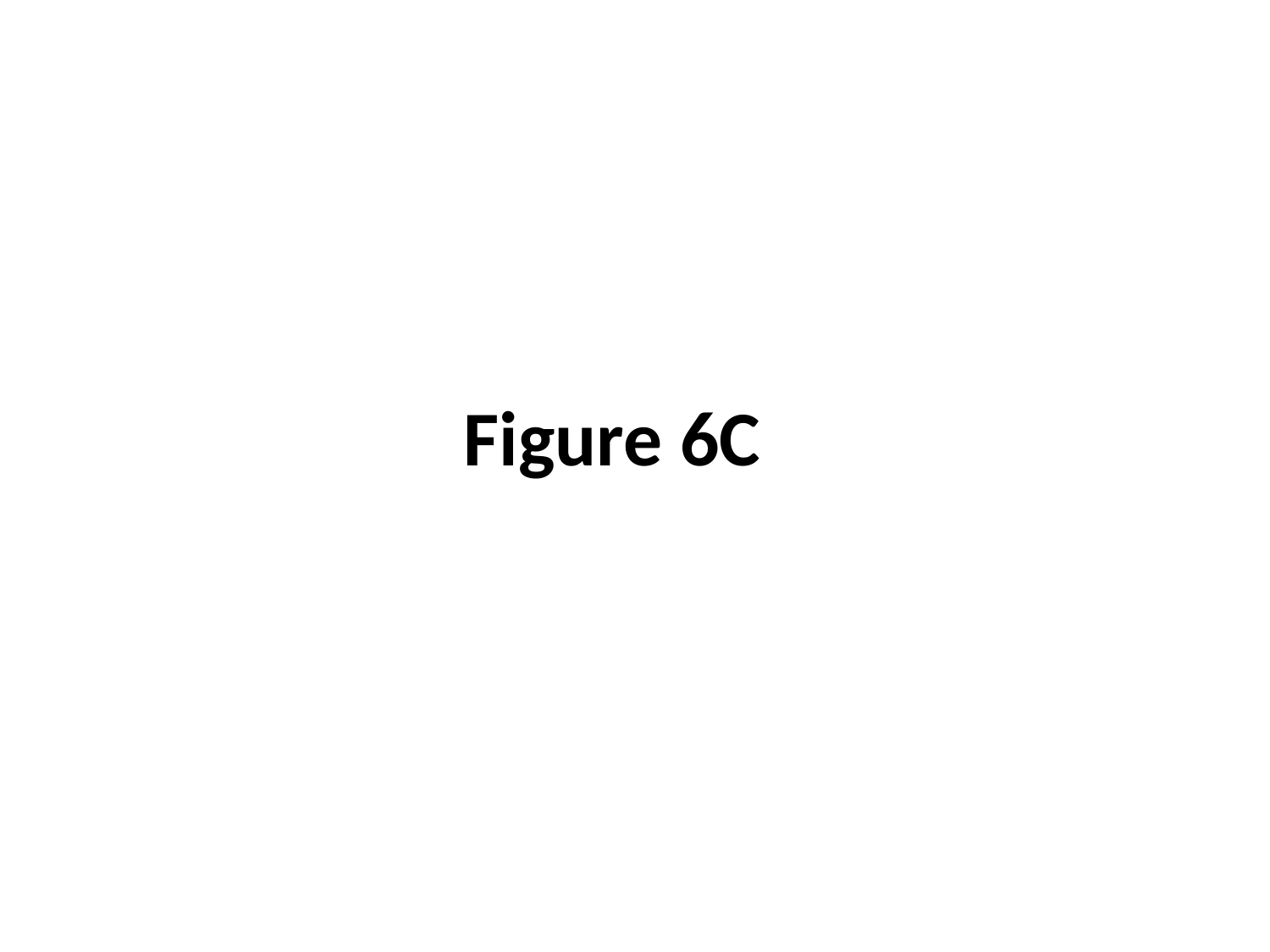

Figure 6C

## Slide 2
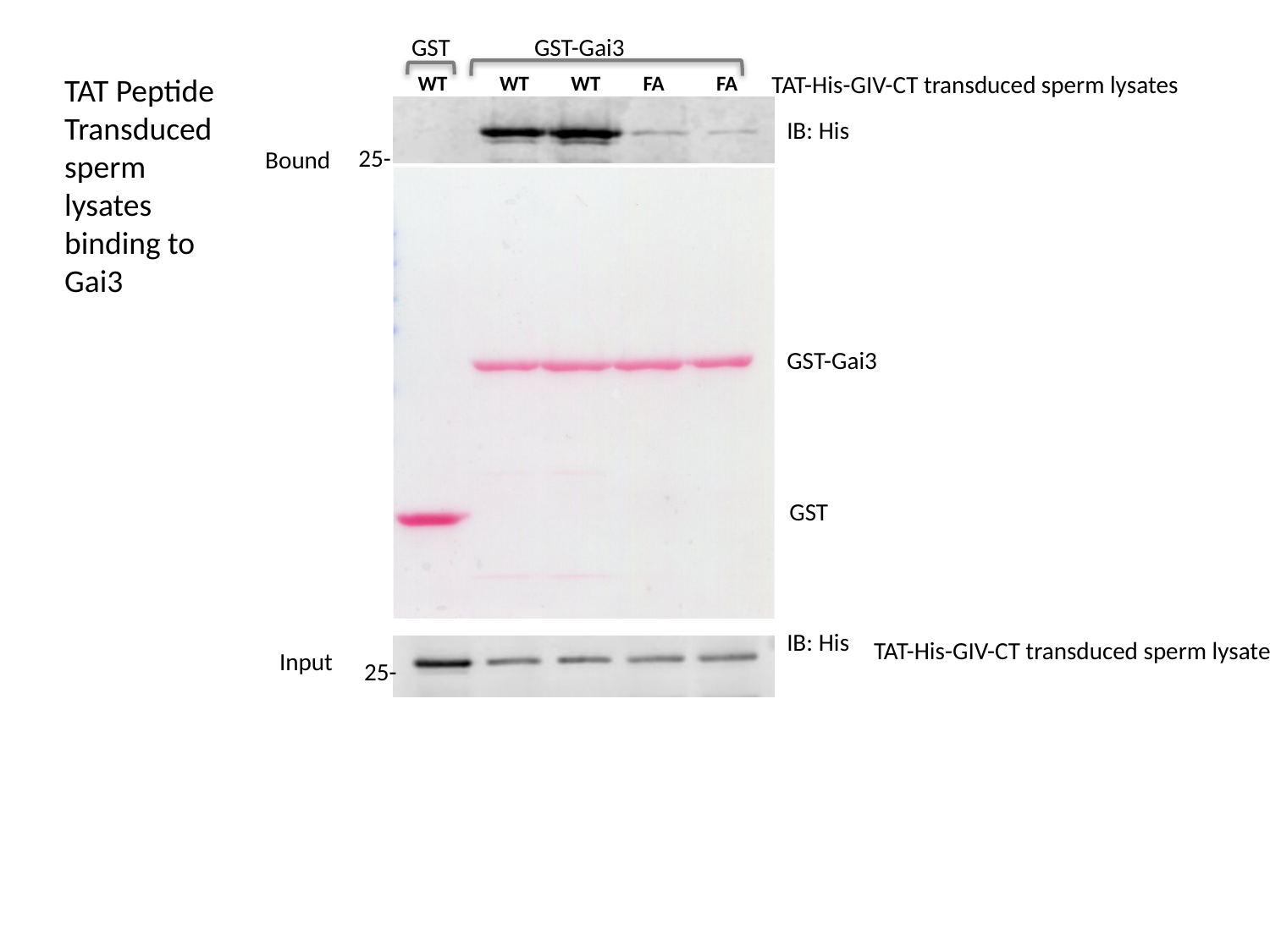

GST
GST-Gai3
TAT-His-GIV-CT transduced sperm lysates
TAT Peptide
Transduced sperm lysates binding to Gai3
WT
WT
WT
FA
FA
IB: His
25-
Bound
GST-Gai3
GST
IB: His
TAT-His-GIV-CT transduced sperm lysates
Input
25-
