# Supplementary material for: GIV/Girdin, a non-receptor modulator for Gαi/s, regulates spatiotemporal signaling during sperm capacitation and is required for male fertility
Source: eLife. 2021 Aug 19;10:e69160. doi: 10.7554/eLife.69160 (PMC8376251; doi:10.7554/eLife.69160)
Supplement: Figure 6—source data 2. [file elife-69160-fig6-data2.pptx]

## Slide 1
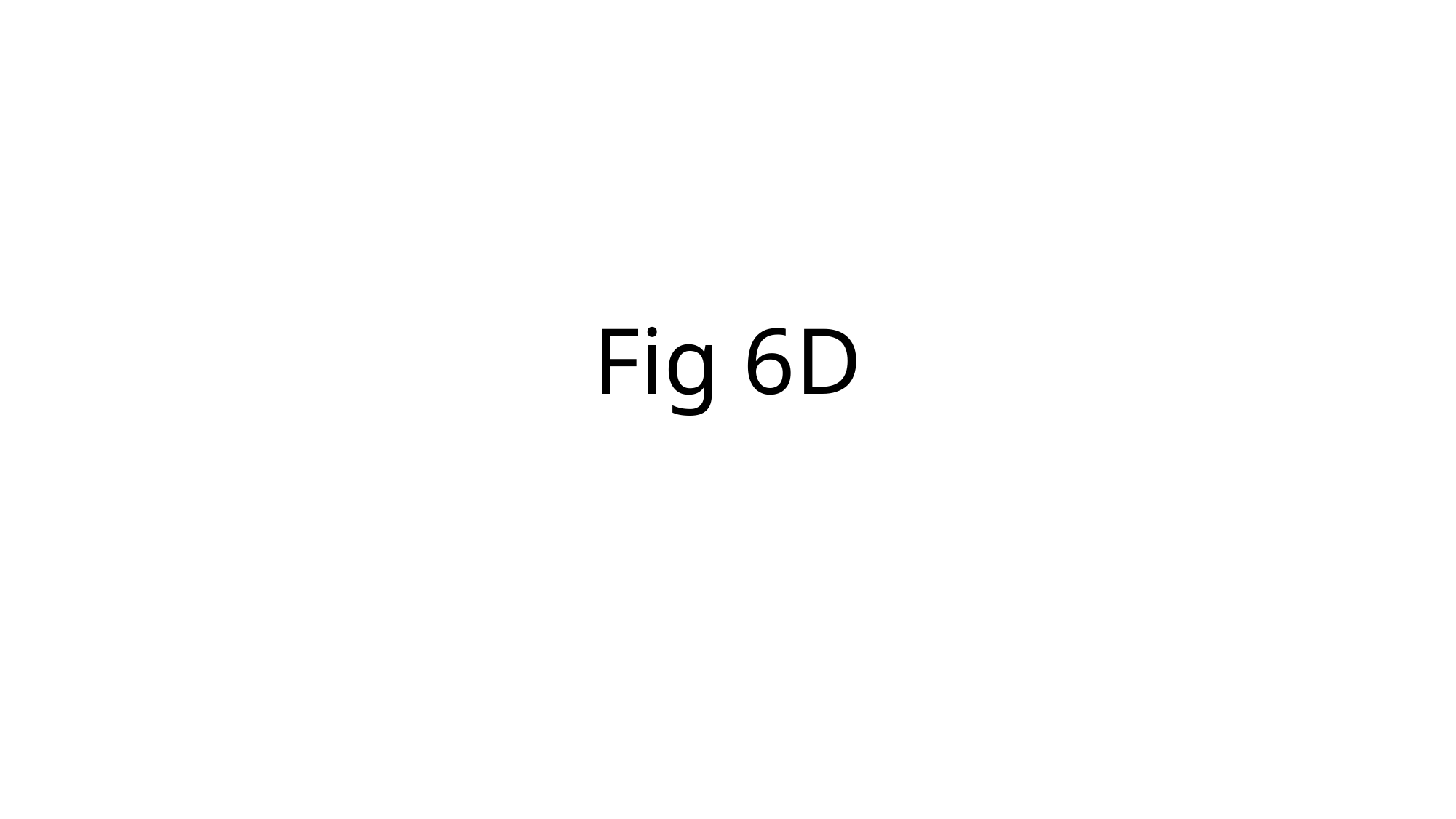

# Fig 6D

## Slide 2
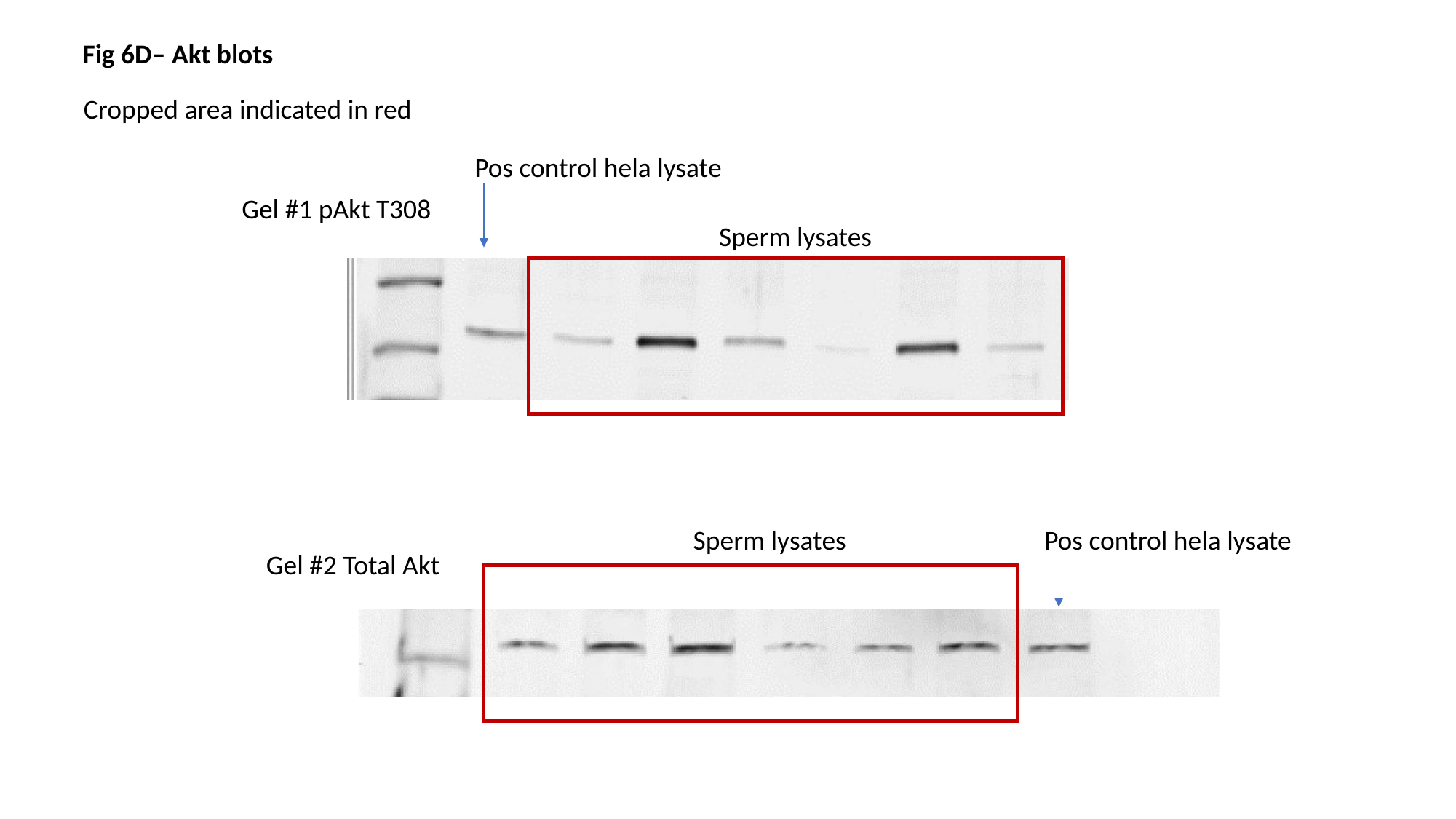

Fig 6D– Akt blots
Cropped area indicated in red
Pos control hela lysate
Gel #1 pAkt T308
Sperm lysates
Sperm lysates
Pos control hela lysate
Gel #2 Total Akt

## Slide 3
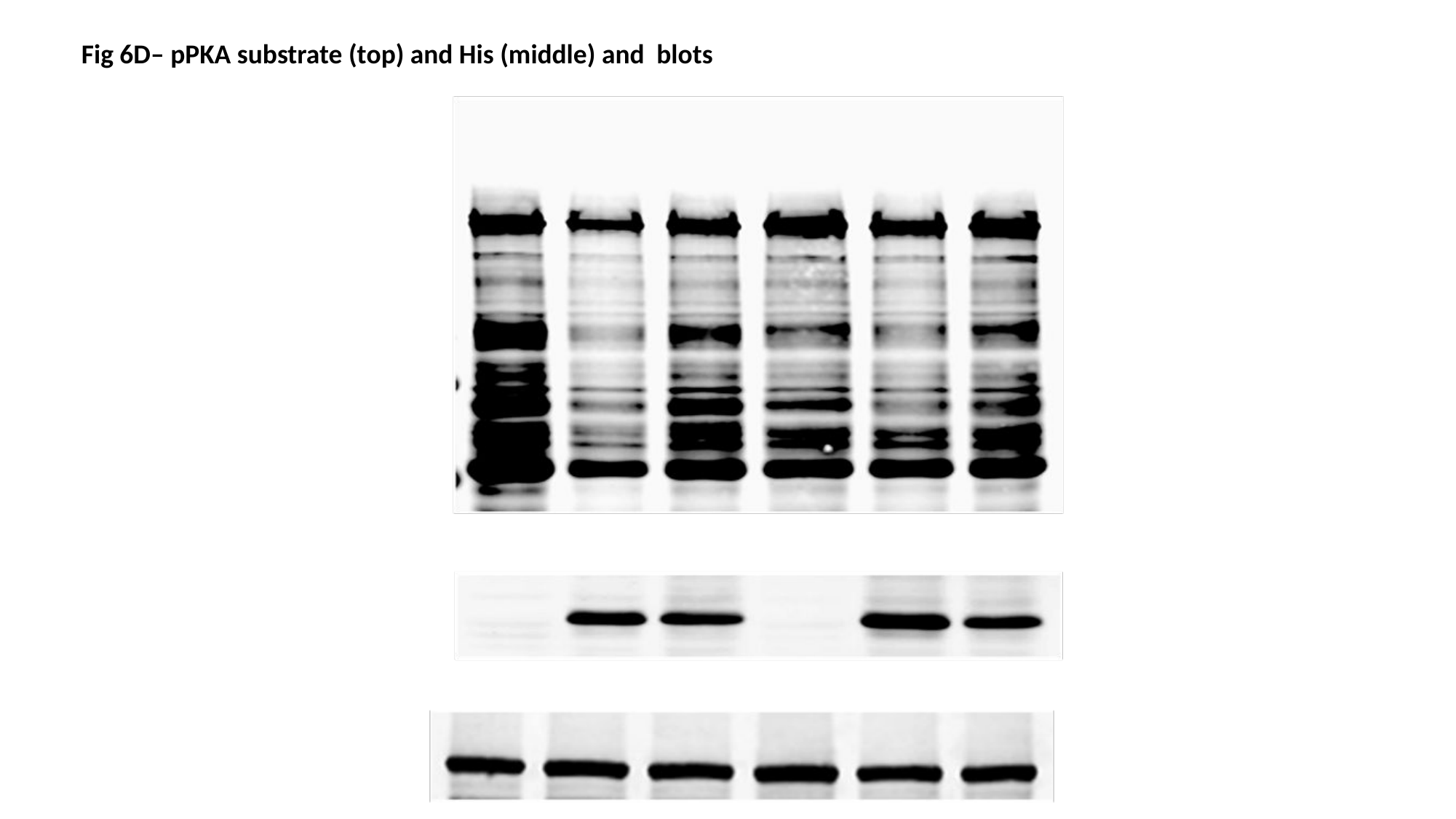

Fig 6D– pPKA substrate (top) and His (middle) and blots
