# Supplementary material for: GIV/Girdin, a non-receptor modulator for Gαi/s, regulates spatiotemporal signaling during sperm capacitation and is required for male fertility
Source: eLife. 2021 Aug 19;10:e69160. doi: 10.7554/eLife.69160 (PMC8376251; doi:10.7554/eLife.69160)

## Slide 1
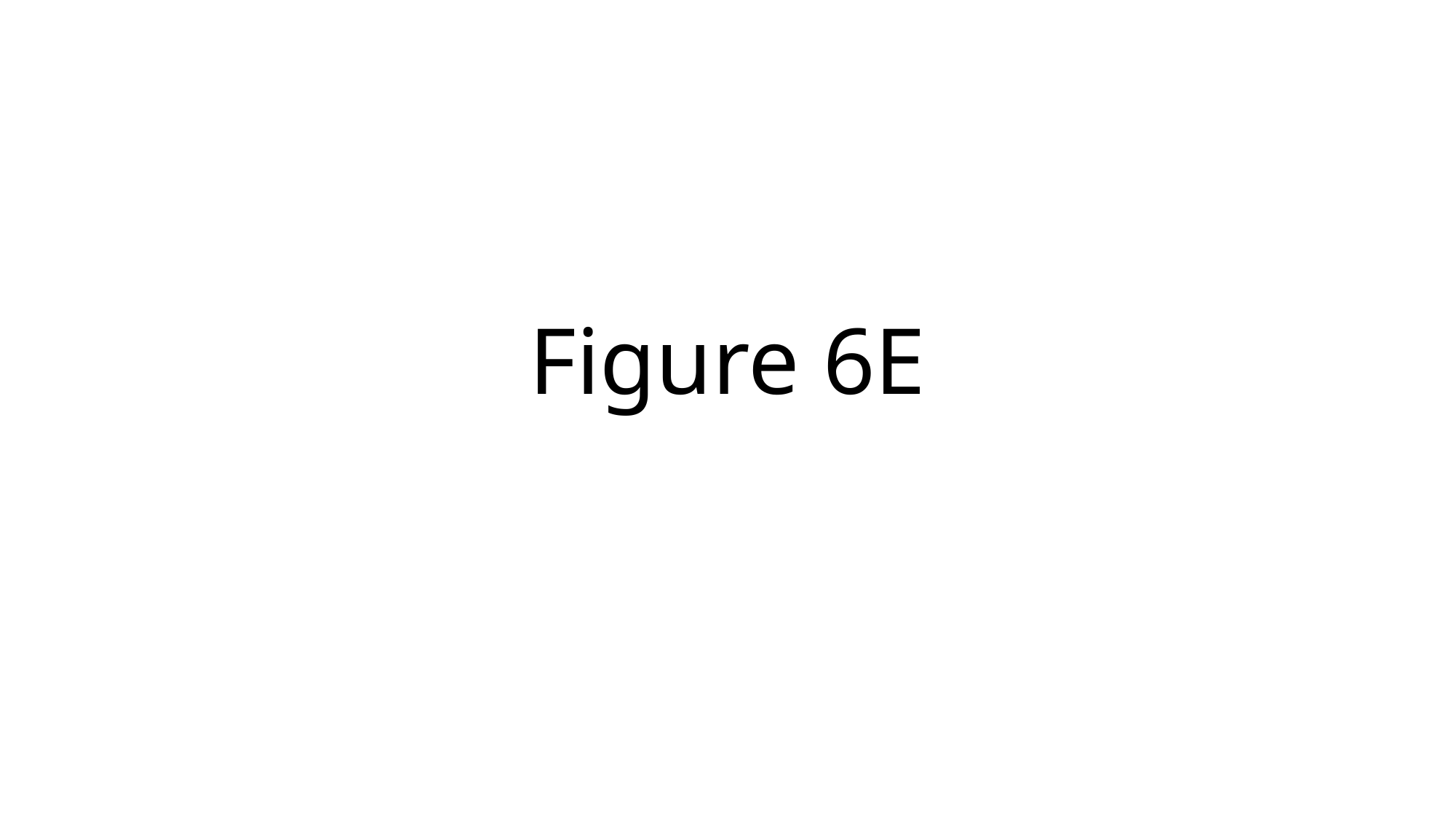

# Figure 6E

## Slide 2
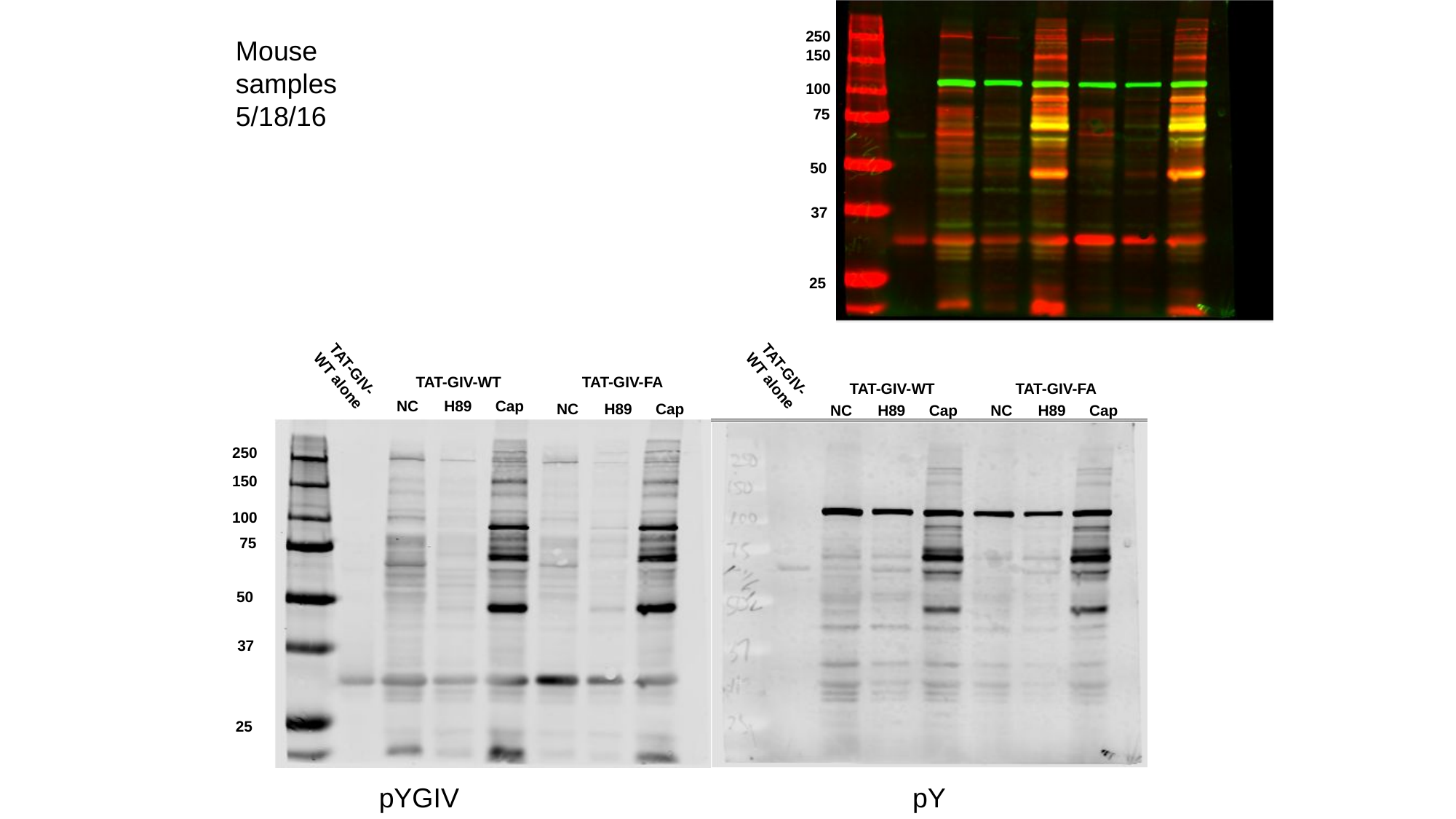

250
Mouse samples
5/18/16
150
100
75
50
37
25
TAT-GIV-WT alone
TAT-GIV-WT alone
TAT-GIV-WT
TAT-GIV-FA
TAT-GIV-WT
TAT-GIV-FA
NC
H89
Cap
NC
H89
Cap
NC
H89
Cap
NC
H89
Cap
250
150
100
75
50
37
25
pYGIV
pY

## Slide 3
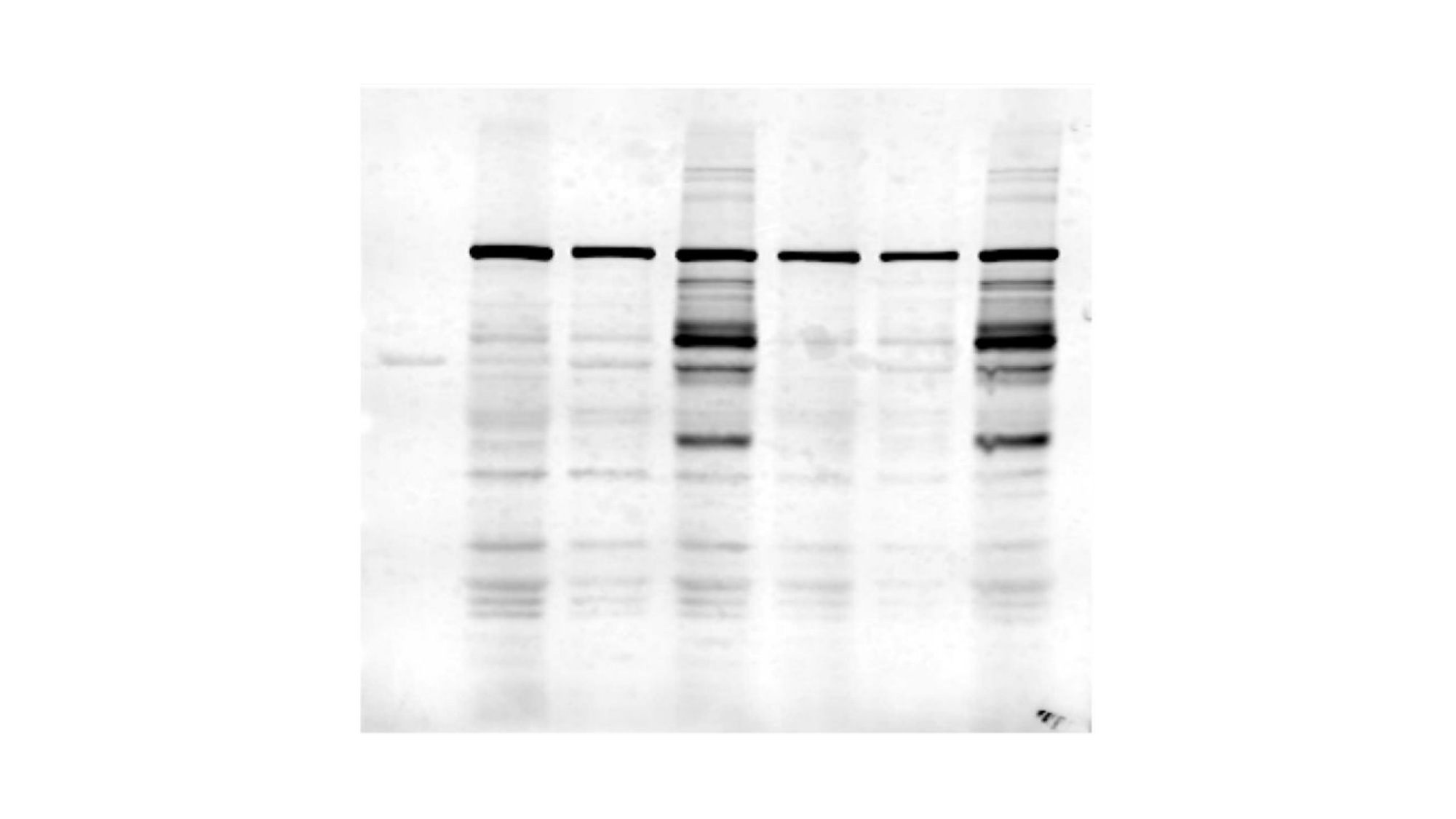

## Slide 4
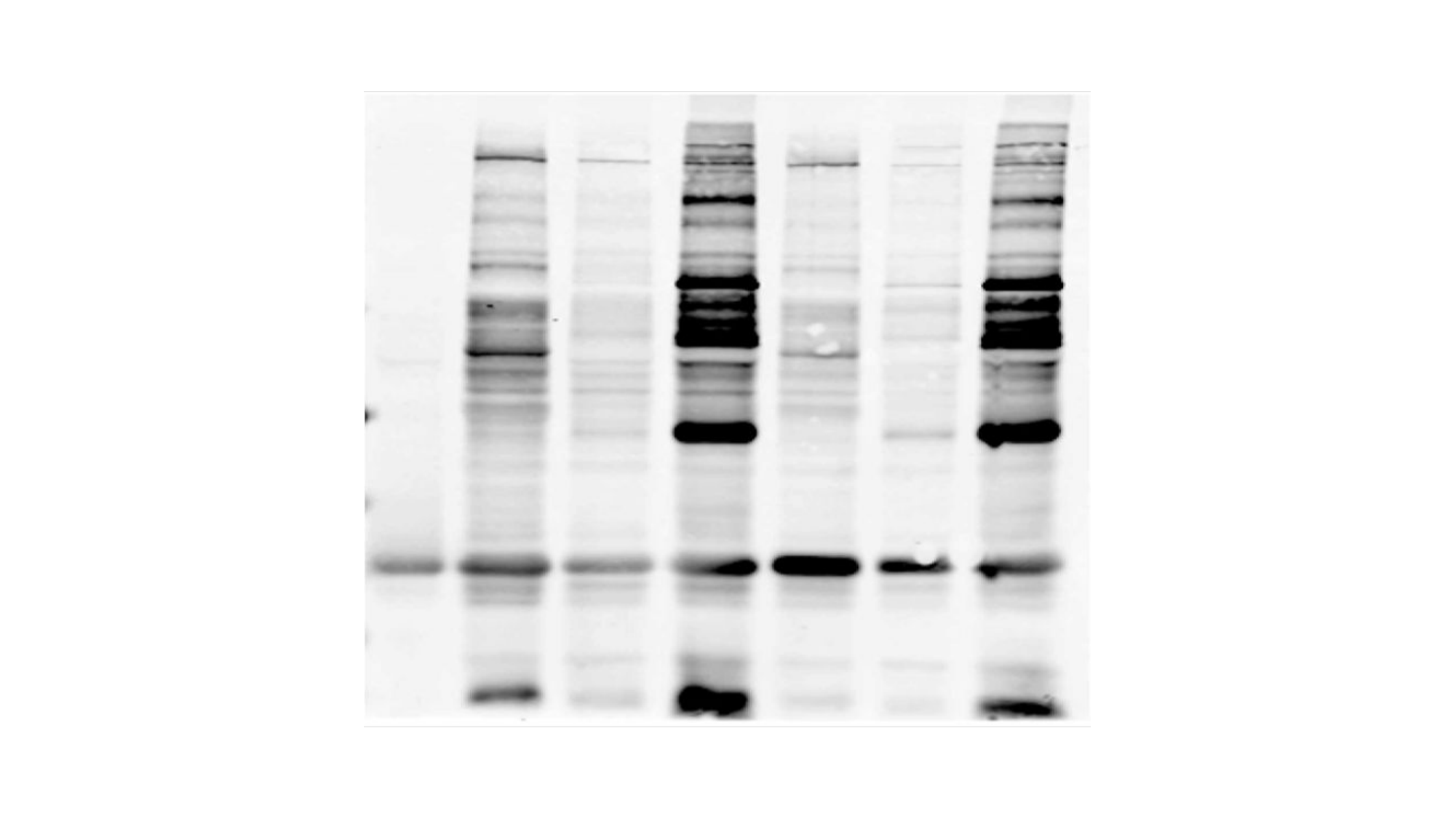

Supplement: Figure 6—source data 3. [file elife-69160-fig6-data3.pptx]
