# Supplementary material for: GIV/Girdin, a non-receptor modulator for Gαi/s, regulates spatiotemporal signaling during sperm capacitation and is required for male fertility
Source: eLife. 2021 Aug 19;10:e69160. doi: 10.7554/eLife.69160 (PMC8376251; doi:10.7554/eLife.69160)
Supplement: Transparent reporting form [file elife-69160-transrepform1.docx]

***eLife’s* transparent reporting form**

**Sample-size estimation**

Sample size and their details are mentioned in the methods and figure legends. Number of biological replicates and technical replicates were decided from the experience, literature survey and to meet enough stastical power. Details of the replicates are described in figure legends. For confocal imaging several fields were imaged without bias and the representative figure has been used in Figure panels. All experiments were performed minimum 3 times and the details are mentioned in methods or in figure legends.

**Replicates**

All biological and technical replicate details are mentioned in the material and methods or Figure legends.

**Statistical reporting**

Statistical significance between datasets with three or more experimental groups was determined using one-way analysis of variance (ANOVA) followed by Tukey’s test for multiple comparisons. Unpaired t-test is used to test the statistical difference between two experimental groups. Data presented as mean±SEM. For all tests, a p-value > 0.05 is considered as significant. All experiments were repeated a least three times. All statistical analysis was performed using GraphPad prism 9. For RNA analysis, mRNA stranded sequencing libraries were generated with the TruSeq Stranded mRNA Sample Prep Kit with TruSeq Unique Dual Indexes (Illumina, San Diego, CA). Resulting libraries were multiplexed and sequenced with 100-bp paired-end reads (PE100) to a depth of approximately 30 million reads per sample on an Illumina NovaSeq 6000. Samples were demultiplexed using bcl2fastq v2.20 Conversion Software (Illumina, San Diego, CA). The details of RNA analysis is described in the ‘material and method’ section.

**Group allocation**

Randomized group allocation was done for the animal experimental groups.

**Additional data files (“source data”)**

RNA seq is uploaded to NCBI GEO [GSE171704].
